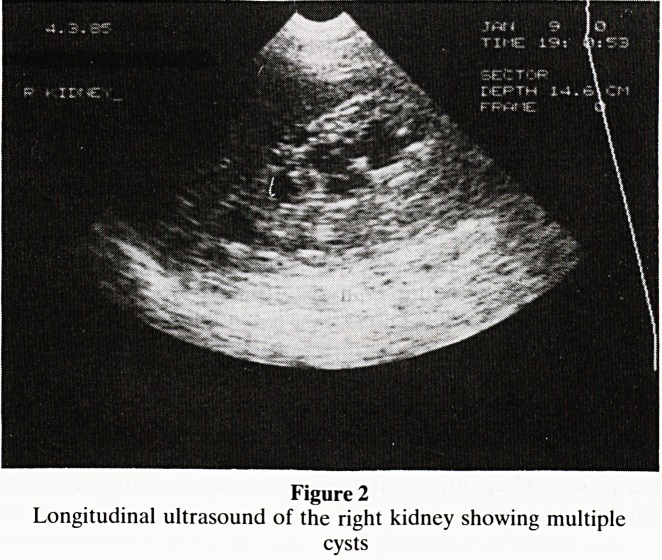# Salutary Lesson in Interpretation of Liver Palpation at Laparotomy for Sigmoid Carcinoma

**Published:** 1988-11

**Authors:** K. R. Malpani, A Hinchcliffe, S. Thompson, P. Goddard

**Affiliations:** Departments of Surgery and Radiology, Weston General Hospital; Departments of Surgery and Radiology, Weston General Hospital; Departments of Surgery and Radiology, Weston General Hospital; Departments of Surgery and Radiology, Weston General Hospital


Bristol Medico-Chirurgical Journal Volume 103 (iv) November 1988

### Salutary Lesson in Interpretation of Liver Palpation at Laparotomy for Sigmoid Carcinoma

**K. R. Malpani, A Hinchcliffe, S. Thompson and P. Goddard**
Departments of Surgery and Radiology, Weston General Hospital

### CASE REPORT

A 46-year-old man was admitted with intestinal obstruction. A barium enema showed an annular carcinoma in the sigmoid colon. Chest X-ray was normal. At operation, in addition to the sigmoid tumour, lymph nodes in the sigmoid mesentery appeared to be invaded by metastases. Both lobes of the liver were palpated and contained numerous hard nodules. The sigmoid colon was resected with its mesentery and lymph nodes, reconstruction was by end-to-end anastomosis with an intubated caecostomy to decompress the proximal colon.

Histology of the tumour showed a well differentiated adenocarcinoma. One mesenteric lymph node contained tumour, three others examined did not. Postoperative liver function tests were normal, except for a low serum albumin.

The patient made a good recovery but a poor prognosis was discussed with the patient and his family.

The patient was seen at six-month intervals and noted to be well. Two years after the operation ultrasound of the abdomen was carried out (figures 1 and 2).

The ultrasound showed that the supposed liver metastases were part of extensive polycystic disease affecting both liver and kidneys. Hypertension and evidence of renal insufficiency had been previously noted and were due to this condition. The renal polycystic disease was confirmed by excretion urography.

### DISCUSSION

Ultrasound is a useful technique for evaluating liver disease and discriminating between metastatic disease, cysts and cirrhosis (1,2).

**Figure 1**
Transverse ultrasound scan of the liver showing several lesions of low echogenicity and distal enhancement. These appearances represent cysts

**Figure 2**
Longitudinal ultrasound of the right kidney showing multiple cysts

If a liver lesion is palpated at surgery it is preferable to see the liver and, if in doubt, to biopsy the lesion. This may not be possible if a lower abdominal incision has been used. In such cases, ultrasound can help to confirm the presence of metastatic disease. Ultrasound has now been incorporated into the routine investigation of many kinds of abdominal malignancy. Normal liver function tests alone do not discredit the diagnosis of hepatic metastases since a number of studies have shown abnormal values in less than 70% of patients with liver secondary deposits (3–6).

### REFERENCES

1. SCHREVE, R. H. TERPSTRA, O. T., AUSEMA, L. *et al.* (1984) Detection of liver metastases, a prospective study comparing liver enzymes, scintigraphy, ultrasonography and computed tomography. *Br.J.Surg.* **71**, 947–9.
2. BERNARDINO, M. E., THOMAS, J. L., BARNES, P. A. *et al.* (1982) Diagnostic approaches to liver and spleen metastases. *The Radiologic Clinics of North America* **20**, 469–86.
3. KEMENY, M. M., SUGARBAKER, P. H. *et al.* (1982) A prospective analysis of laboratory tests and imaging studies to detect hepatic lesions. *Ann.Surg.* **195**, 163–167.
4. TARTTER, P. I., SLATER, G., GELERNT, I. *et al.* (1981) Screening for liver metastases from colorectal cancer with carcino-embyonic antigen and alkaline phosphatase. *Ann.Surg.* **193**, 357–60.
5. WATSON, A. and TORRANCE, H. B. (1969) Liver scanning in surgical practice. *Br.J.Surg.* **57**, 405–8.
6. OXLEY, E. M., and ELLIS H. (1969) Prognosis of carcinoma of the large bowel in the presence of liver metastases. *Br.J.Surg.* **56**, 149–52.

## Figures and Tables

**Figure 1 f1:**
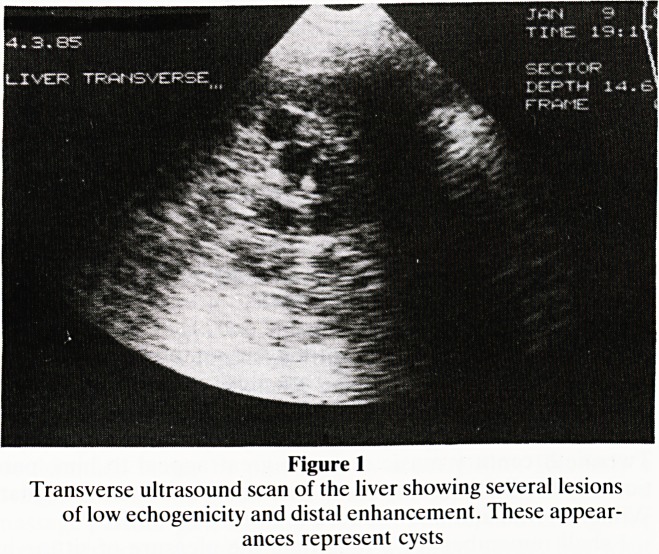


**Figure 2 f2:**